# New Medical Journals

**Published:** 1850-03

**Authors:** 


### ARTICLE II.

### NEW MEDICAL JOURNALS.

*Transylvania Medical Journal*—Edited by E. L. DUDLEY, M.D., Prof. of Gen. and Path., Anatomy and Physiology, under the supervision of the Transylvania Faculty of Medicine.

We have lately received, in exchange, the back numbers of this new candidate for favor at the hands of the Medical public in the West, and welcome it to our list. Issuing from the oldest and one of the most respectable Medical Institutions in the West, it ought not fail to receive that liberal support that will enable its proprietors ably to sustain the enterprise.—We find many highly interesting articles in its pages, and shall gladly avail ourselves of the opportunity of enriching our pages by extracts from them, from time to time. It is published every other month, each number containing ninety-six pages, at three dollars per annum in advance.

*The St. Louis Probe*—Edited by A. J. COONS, M.D., and JOHN R. ATKINSON, M.D.

We have received in exchange two numbers of this work. The Probe is issued monthly, each number containing twenty-four pages, at two dollars per annum, with a promise, that if sufficient patronage is given it, to justify the expense, it will be issued every two weeks at the same price.

We observe by the prospectus that it is to be "devoted to the dissemination of the latest Medical Intelligence, and to the investigation and exposure of the numerous errors and abuses which unhappily prevail in the Profession of Medicine."

We observe also, that the prospectus says:—

"While we shall not cater to the opinions of general readers, it shall be our purpose to insert such matter in the Probe as to render it interesting to that class; for we hold that the only way to protect the community from the impositions of charlatans and pretenders in medicine, is to lay before them information from which they may deduct rules that will enable them to detect imposture in whatever garb it may be presented to them."

Now while we welcome back to the editorial fraternity Dr. Coons, formerly one of the editors of the Missouri Medical Journal, and welcome Dr. Atkinson fresh into the field of labor, we cannot help pointing out what we conceive to be an objection to the plan they propose their work to pursue. We mean its attempted adaptation to the professional and the popular reader. We have no objection to "the investigation and exposure of the numerous errors and abuses" of the profession; nor to the publication of such matter as will enlighten public opinion in reference to the profession; but we do see serious objections to an attempt to improve the profession by a journal designed for popular circulation.

We submit whether it will be likely to promote the respectability, good standing, or usefulness of the profession to discuss in a popular paper, the errors and abuses that unhappily prevail in it, when from the very nature of those errors and abuses they will often necessarily be misunderstood by the public. Nor can we conceive how a Scientific Medical Journal, which shall be instructive and interesting to the Physician, can be made up of such matter, as to be interesting to the general reader. We opine our friends will find it necessary to apply their Probe exclusively, either to the professional or popular wounds. E.

